# Effects of automated alerts on unnecessarily repeated serology tests in a cardiovascular surgery department: a time series analysis

**DOI:** 10.1186/1472-6963-10-70

**Published:** 2010-03-19

**Authors:** Julie Niès, Isabelle Colombet, Eric Zapletal, Florence Gillaizeau, Patrick Chevalier, Pierre Durieux

**Affiliations:** 1 Centre de Recherche des Cordeliers, INSERM, U872 Eq. 20, Paris, F-75006 France; Université Pierre et Marie Curie - Paris6, UMR S 872, Paris, F-75006 France; Université Paris Descartes, UMR S 872, Paris, F-75006 France; 2 Medical Informatics Department, Georges Pompidou European Hospital, 75015 Paris, France; 3 MEDASYS, Espace technologique de St Aubin, 91193 Gif-sur-Yvette Cedex, France; 4 Cardio-Vascular Surgery Service, Georges Pompidou European Hospital, 75015 Paris, France

## Abstract

**Background:**

Laboratory testing is frequently unnecessary, particularly repetitive testing. Among the interventions proposed to reduce unnecessary testing, Computerized Decision Support Systems (CDSS) have been shown to be effective, but their impact depends on their technical characteristics. The objective of the study was to evaluate the impact of a Serology-CDSS providing point of care reminders of previous existing serology results, embedded in a Computerized Physician Order Entry at a university teaching hospital in Paris, France.

**Methods:**

A CDSS was implemented in the Cardiovascular Surgery department of the hospital in order to decrease inappropriate repetitions of viral serology tests (HBV).

A time series analysis was performed to assess the impact of the alert on physicians' practices. The study took place between January 2004 and December 2007. The primary outcome was the proportion of unnecessarily repeated HBs antigen tests over the periods of the study. A test was considered unnecessary when it was ordered within 90 days after a previous test for the same patient. A secondary outcome was the proportion of potentially unnecessary HBs antigen test orders cancelled after an alert display.

**Results:**

In the pre-intervention period, 3,480 viral serology tests were ordered, of which 538 (15.5%) were unnecessarily repeated. During the intervention period, of the 2,095 HBs antigen tests performed, 330 unnecessary repetitions (15.8%) were observed. Before the intervention, the mean proportion of unnecessarily repeated HBs antigen tests increased by 0.4% per month (absolute increase, 95% CI 0.2% to 0.6%, *p *< 0.001). After the intervention, a significant trend change occurred, with a monthly difference estimated at -0.4% (95% CI -0.7% to -0.1%, *p *= 0.02) resulting in a stable proportion of unnecessarily repeated HBs antigen tests. A total of 380 unnecessary tests were ordered among 500 alerts displayed (compliance rate 24%).

**Conclusions:**

The proportion of unnecessarily repeated tests immediately dropped after CDSS implementation and remained stable, contrasting with the significant continuous increase observed before. The compliance rate confirmed the effect of the alerts. It is necessary to continue experimentation with dedicated systems in order to improve understanding of the diversity of CDSS and their impact on clinical practice.

## Background

Clinical Decision Support Systems (CDSS) are defined as information systems designed to improve clinical decision making. They have demonstrated their efficacy in improving clinical practices and patient outcomes [1-7], particularly under the form of on-screen computer reminders [8]. CDSS are recommended to healthcare organizations, especially those integrated into Hospital Information Systems (HIS) [9]. An entirely computerized HIS is made of several components, including an Electronic Health Record (EHR), a Computerized Physician Order Entry (CPOE), and radiology, laboratory and pharmacy information subsystems. The capacity of a CPOE to provide alerts and decision support to the physician constitutes one of the characteristics included in the evaluation of its quality [10].

Laboratory testing of hospitalized patients can sometimes be unnecessary, particularly repetitive testing [11]. The CPOE itself can favor such repetitive testing, particularly when its workflow does not correspond to the usual behavior of the physician (eg, when a physician orders a test for a patient and does not know that the same test has already been recently ordered, even if the result is stored in the CPOE database) [12]. However, CPOE with embedded decision-support tools could reduce such repetitive testing [11,13,14]. In this study, we hypothesized that a reminder of previous existing results (collected from all available patient stays) could prevent unnecessary viral serology testing.

We evaluated the impact of a Serology-CDSS providing point of care reminders of previous existing serology results, embedded in a CPOE used in a university teaching hospital.

## Methods

### Study site

The study was performed in the Cardiovascular Surgery department of the Georges Pompidou European Hospital (HEGP), a university teaching hospital in Paris, France. The hospital has 806 beds organized around 3 major medical departments: cardiovascular, cancer and internal medicine, and an emergency center. There are approximately 204,000 consultations and 55,000 admissions per year. Cardiac surgery and transplantation constitute an important part of its activity.

Since opening in 2000, the hospital has used an entirely computerized HIS centered on DxCare^® ^an industry EHR [15]. The EHR facilitates the computerized prescription of drugs, imaging and laboratory tests by means of a CPOE. Every test order is made through the CPOE by a physician on the house staff. The order is immediately visible on the nursing care plan. It is transmitted in parallel to the laboratory software, which records that a laboratory sample will be sent to the lab. DxCare^® ^also permits the viewing of laboratory results. By default, the results shown are those for the current patient stay in the hospital, which means that a physician who wants information from previous stays has to actively search for it. During year 2005, among the 1669 viral serology tests ordered for patients hospitalized in the cardiovascular surgery department, 280 (17%) were ordered within 90 days after a previous such test for the same patient and were considered unnecessary by the resource utilization committee of the hospital.

### Viral serology testing

In this study, viral serology testing is defined in screening protocols for hepatitis B virus (HBV), associated or not with hepatitis C virus (HCV). Serological tests for HBV, either in isolation or with other viral serology tests, are the most frequently ordered viral serology tests. The protocol for HBV testing involves the simultaneous prescription of three individual tests; only one of those, the test for HBs antigen, is carried out systematically. The other two tests, for antibodies against HBs and HBc, are carried out only if the HBs antigen is detected. The evaluation was therefore solely based on the data analysis of HBV tests, and especially HBs antigen tests.

The resource utilization committee of the hospital defined a serology test as unnecessarily repeated if the order occurred within 90 days from the previous result. The 90 day period was chosen because it corresponds to the mean incubation period of the disease.

### Intervention

**Figure 1:**
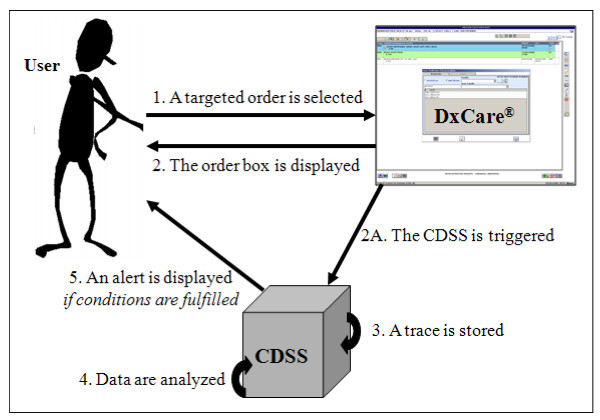
**Overview of the CDSS integrated with DxCare**. The CDSS automatically prompts the physician with previous existing results, when he/she initiates the order of a potentially redundant test. The system records the intervention in order to facilitate its evaluation.

**Figure 2:**
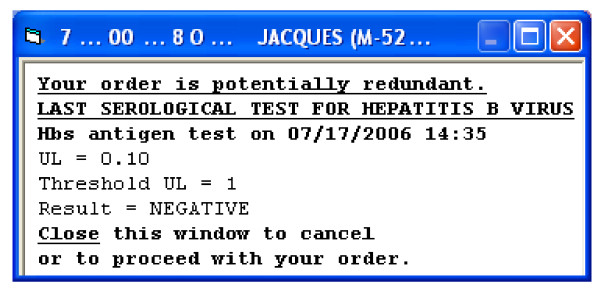
**Example of an alert displayed for a potentially unnecessary HBV test order**. The alert content has been translated from French.

The Serology-CDSS is triggered when one of the targeted serological tests for HBV is selected to be ordered (Figure 1 - steps 1 & 2A). The Serology-CDSS stores a record of its execution each time a physician selects a viral serology test order (Figure 1 - step 3), ie, each time the physician intends to order that test. The system takes into account the most recent laboratory results for viral serology tests listed in the patient's EHR and their dates, regardless of their relationship to the current stay (Figure 1 - step 4). An alert is displayed if the most recent result of the targeted laboratory test for the patient is less than 90 days old (Figure 1 - step 5). Conversely, if no result is available or if the last available result was more than 90 days old, no alert is displayed. The Serology-CDSS displays a new HTML page concomitant with the order entry window (Figure 1 - steps 2 & 5). The alert informs the user of the date and complete result of the last serological test carried out for the patient (Figure 2). The alert is displayed only to prescribers who are members of the house staff.

The Serology-CDSS has been developed to include four technical characteristics known to be associated with decision support systems success [3,4,16]: the alert was automatically prompted and was part of clinician workflow, the user could not deactivate the alert output, the most recent laboratory result for viral serology tests and its date was automatically retrieved from the patient's EHR, and the alert was displayed at the time and location of decision making (ie, before the user ordered an unnecessarily repeated test).

### Study design

We evaluated the effect of the intervention by analyzing the time series of the monthly proportion of repeated HBs antigen tests [17]. The Serology-CDSS became functional in the Cardiovascular Surgery department in June 2006; several modifications were made up to the end of November 2006. As recommended [18-20], we collected data from 2004 to 2007 to have sufficient points before and after the start of the intervention to account for seasonally correlated errors. June 2006 was excluded from analysis because the intervention was initiated mid-month. Three periods were defined: the pre-intervention period (January 2004 to May 2006), the adjustment period (July 2006 to November 2006) and the effective intervention period (December 2006 to December 2007).

### Outcome measures

#### Primary outcome

The primary outcome was the proportion of unnecessary repetitions of viral serology tests. A test was considered unnecessary when it was ordered within 90 days after the presence in the EHR of a result of the same test for the same patient. The test could have been ordered in the Cardiovascular Surgery department or in another department. Conversely, HBs antigen tests were not considered as unnecessarily repeated when previous results were unavailable (poor sample, broken tube...). Laboratory results of interest were extracted from the DxCare^® ^database for the study period.

#### Secondary outcome

Each time a physician intended to order a test within the 90 day limit, a record was stored in the Serology-CDSS database. The records stored by the Serology-CDSS make it possible to estimate the proportion of all intentions to repeat a test order within 90 days of the last test. Compliance with the alert was evaluated by comparing the number of HBV tests unnecessarily repeated, ie, the number of 90-day repeated results compared to the number of potentially unnecessary HBV test orders, ie, the number of « alerts displayed ».

### Statistical analysis

We used a segmented regression analysis to determine the impact of the intervention, both immediately (change in level) as well as over time (change in trend) [18,21]. The full segmented regression model included a baseline trend, a change in level and trend after the start of the intervention (July 2006), and a change in level and trend after the adjustment period (December 2006). The most parsimonious model was obtained after eliminating non-significant terms using a backward selection. Our final model did not include correction for seasonal variation (not detected), nor adjustment for autocorrelation (residuals were independent, normally distributed with mean zero and constant variance). In a similar way, we compared the change in level and trend between periods for characteristics of patients (sex ratio, age) and hospital stays (frequency of stays with transplant, length of stay) using four segmented regression models (time series data not shown). We identified patients and hospital stays for a period according to the first HBs antigen test in the Cardiovascular Surgery department.

SAS statistical software (release 9.1; SAS Institute Inc., Cary, NC, USA) was used for all analyses.

According to French laws, this study did not required to be approved by an ethical committee, since it concerned the improvement of quality of care and routine practice.

## Results

### Characteristics of patients and hospital stays

During the study period (June 2006 excluded) 4,415 patients had at least one HBs antigen test in the Cardiovascular Surgery department (5,412 hospital stays). Characteristics of patients and hospital stays according to period are presented in table 1. 3,132 (70.9%) patients were male and the median age was 64 (interquartile range [IQR], 53 to 74). 85 (1.6%) hospital stays concerned transplanted patients and median length of stay was 10 days (IQR, 5 to 16). According to the segmented regression analysis, the sex ratio and median age of patients did not change during the study period (no significant level or trend change between the three periods). However, there was an increase of stays with transplant during the pre-intervention period and a significant decrease of length of stay during the effective intervention period (trend change).

**Table 1:** Characteristics of patients and hospital stays with at least one HBs antigen test in the Cardiovascular Surgery department during the study period.

Period*†	Pre-intervention	Intervention period	
		
Characteristics	period	Adjustment period	Effective intervention period
**Patients**‡	**(n = 2888)**	**(n = 455)**	**(n = 1072)**
Male, n (%)	2044 (70.8)	321 (70.5)	767 (71.5)
Age (years), median (IQR)	64 (53 to 74)	64 (54 to 74)	65 (55 to 74)
			
**Hospital stays**§	**(n = 3412)**	**(n = 605)**	**(n = 1395)**
With transplant, n (%)	34 (1.0)	16 (2.6)	35 (2.5)
Length of stays (days), median (IQR)	10 (4 to 15)	10 (4 to 15)	10 (5 to 16)

IQR = Interquartile Range

* We identified patients and hospital stays for a period according to the first HBs antigen test in the Cardiovascular Surgery department.

† Pre-intervention period: January 2004 to May 2006, Adjustment period: July 2006 to November 2006; Effective intervention period: December 2006 to December 2007. June 2006 is not presented: it has been excluded from analysis since the intervention was initiated mid-month. 117 patients (157 hospital stays) had a first HBs antigen test in the Cardiovascular Surgery department during June 2006.

‡ The two segmented regression models predicting monthly proportion of males and median age of patients indicated there were no level and trend changes for age and sex ratio during the study period (time series data not shown).

§ The two segmented regression models predicting monthly proportion of stays with transplant and median length of stays indicated respectively an increase of stays with transplant during the pre-intervention period and a significant decrease of length of stays during the effective intervention period (trend change) (time series data not shown).

### Impact of the viral serology alert

A total of 5,575 HBs antigen tests were ordered in the Cardiovascular Surgery department during the study (June 2006 excluded). In the pre-intervention period, 3,480 tests were ordered, of which 538 (15.5%) were unnecessarily repeated. For 11.2% of them (60/538), at least three such tests were ordered for the patient. During the intervention period, 2,095 HBs antigen tests were performed, with 330 unnecessary repetitions (15.8%): 99 (15.6%) when the CDSS was in the adjustment period and 231 (15.8%) during the effective intervention period. 14.6% of the repeated tests (48/330) were the third or more test ordered for the patient.

Among the 868 unnecessarily repeated tests, 865 were done when previous serology test result was negative and 3 when the result was positive. The percentage of accepted messages was higher when the previous serology test result was positive: 86.4% (19/22) versus 48.2% when the previous test was negative (805/1670) (p < 0.001).

During the study period, 31.0% (27/87) of patients with hospital stays for transplant had an unnecessary repetition of viral serology tests versus 15.4% (844/5482) for other stays. In the pre-intervention and intervention periods respectively, 10 of 538 (1.9%) and 23 of 330 (7.0%) unnecessarily repeated tests occurred for transplant stays.

**Figure 3:**
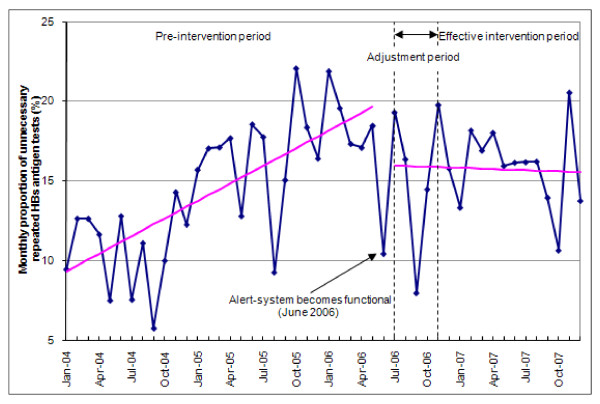
**Changes in the repetition of HBs antigen testing from January 2004 to December 2007**. A segmented regression analysis was performed to determine the impact of the intervention. Three periods were defined: the pre-intervention period (January 2004 to May 2006), the adjustment period, corresponding to CDSS adjustments (July 2006 to November 2006) and the effective intervention period (December 2006 to December 2007). June 2006 has been excluded from analysis since the intervention started mid-month.

Figure 3 shows the time series of the proportions of unnecessarily repeated HBs antigen tests from January 2004 to December 2007. Segmented regression analysis revealed a significant month-to-month change before the beginning of the CDSS integration. Before June 2006, the mean proportion of unnecessarily repeated HBs antigen tests was estimated at 9.0% per month (95% CI 6.6% to 11.3%) and increased by 0.4% per month (absolute increase, 95% CI 0.2% to 0.6%, *p *< 0.001).

There were no significant differences in level or in trend change between the two sub-periods of the intervention (adjustment period and effective intervention period). In June 2006, the estimated mean proportion of HBs antigen tests dropped by 4%. A significant trend change occurred with a monthly difference of -0.4% (95% CI -0.7% to -0.1%, *p *= 0.02) compared to the trend of the pre-intervention period. This resulted in a stable unnecessarily repeated test rate during the intervention period (non-significant reduction of 0.02%, *p *= 0.87).

### Compliance with the alert

During the intervention period (adjustment period and effective intervention period), the Serology-CDSS was triggered 7,342 times for all serological test orders combined and 2,600 times for HBV test orders. Five hundred alerts were displayed for potentially redundant HBV tests, and 380 unnecessary tests were ordered despite the alerts (compliance rate 24%).

## Discussion

Despite the fact that the mean proportions of unnecessarily repeated test ordering were quite similar before and after the intervention, the time series analysis showed that the proportion of unnecessarily repeated tests dropped by 4% and a significant trend change occurred after the Serology-CDSS introduction, with a stable rate contrasting with the continuous increase observed before (difference in the monthly trend estimated at -0.4%). Compliance rate confirmed the effect of the alerts: 24% of unnecessary test orders were cancelled in the intervention period.

In addition, the percentage of accepted messages was higher when the previous serology test result was positive.

A range of interventions has been proposed to reduce inappropriate use of laboratory services, including reminders and policies restricting ordering of laboratory tests (such as changes in test request forms or ordering procedures) [11,13,14]. On-screen, computer reminders have been shown to be the most effective [8]. Bates *et al. *in a randomized controlled trial showed that computerized reminders for redundant tests was effective, and appeared to result in no loss of clinical information [13]. Neilson *et al.*, in an interrupted time series, studied two interventions [11]. First, a daily prompt was implemented to ask the providers whether they wanted to discontinue tests scheduled beyond 72 hours. In a second intervention, testing options were constrained by unbundling serum metabolic panel tests into single components and reducing the ease of repeating targeted tests. The first intervention decreased orders by 24% and the second intervention produced an additional decrease of 51%. This dramatic improvement could be explained by the high constraints put on physician behavior; the physician could not easily override the intervention, which was not the case in our study. However, most of the high-quality literature regarding health information technology systems comes from four benchmark North American research institutions [4]. Our study provides interesting information on the effects of a CDSS implemented in a multifunctional commercially developed system for a European institution.

Randomized control trials are the gold standard for evaluating health care interventions. However, time series analysis can be used when it is difficult to randomize. The multiple time points before the intervention, as was done in our study, are considered to be the most important influence on the analysis technique when analyzing any cyclical (seasonal) effect [20].

Our study has some limitations, and it is difficult to explain why messages were overridden. First, it is possible that, despite the message, some physicians decided to order the test because the patient presented new symptoms which could be associated with the occurrence of hepatitis. The fact that the percentage of accepted messages was higher when the previous serology test result was positive could favor this hypothesis. Viral serology tests could also have been periodically ordered according to predefined protocols, especially for transplanted patients. However, the number of transplanted patients is not high enough to explain the remaining repeated viral serology tests.

It is also possible that the physicians did not consider the message important enough to follow because it was not based on published evidence. In other words, they could have thought that such a message might interact negatively with their own clinical judgment.

Second, the compliance rate was evaluated on the basis that the selection of an exam corresponded to an ordering intention. However, the ordering (or the prescribing) process is often interrupted (eg, by phone calls). This could lead to a user logout because of the inactive session. When the user logs in again, he selects the exam to be ordered again, and the Serology-CDSS is triggered twice for a single ordering intention. We have no data to support this hypothesis.

## Conclusion

In conclusion, the proportion of unnecessarily repeated tests immediately dropped after CDSS implementation and remained stable, contrasting with the significant continuous increase observed before, confirming the effect of the alerts. Our CDSS has taken into account the four technical characteristics known to influence its impact on medical practice [3,4,16], especially the integration into clinical workflow. Our study confirms that those characteristics are not sufficient to guarantee a large CDSS impact [4,8,22]. Future research attempting to change physician behavior should incorporate relevant behavior change models [23], in order to better adapt interventions to physician practice.

## Competing interests

The authors declare that they have no competing interests.

## Authors' contributions

All authors contributed to writing the manuscript. IC, PC and PD defined the alert functionality and the evaluation schema. JN and EZ created the alerts and the CDSS design. FG provided the statistical analysis. All authors read and approved the final manuscript.

## Pre-publication history

The pre-publication history for this paper can be accessed here:

http://www.biomedcentral.com/1472-6963/10/70/prepub

## References

[B1] Mollon B Chong JJ Holbrook AM Sung M Thabane L Foster G Features predicting the success of computerized decision support for prescribing: a systematic review of randomized controlled trials BMC Med Inform Decis Mak 2009 9 1 11 10.1186/1472-6947-9-11 19210782 PMC2667396

[B2] Pearson SA Moxey A Robertson J Hains I Williamson M Reeve J Newby D Do computerised clinical decision support systems for prescribing change practice? A systematic review of the literature (1990-2007) BMC Health Serv Res 2009 9 1 154 10.1186/1472-6963-9-154 19715591 PMC2744674

[B3] Kawamoto K Houlihan CA Balas EA Lobach DF Improving clinical practice using clinical decision support systems: a systematic review of trials to identify features critical to success Bmj 2005 330 7494 765 10.1136/bmj.38398.500764.8F 15767266 PMC555881

[B4] Garg AX Adhikari NK McDonald H Rosas-Arellano MP Devereaux PJ Beyene J Sam J Haynes RB Effects of computerized clinical decision support systems on practitioner performance and patient outcomes: a systematic review Jama 2005 293 10 1223 1238 10.1001/jama.293.10.1223 15755945

[B5] Johnston ME Langton KB Haynes RB Mathieu A Effects of computer-based clinical decision support systems on clinician performance and patient outcome. A critical appraisal of research Ann Intern Med 1994 120 2 135 142 8256973 10.7326/0003-4819-120-2-199401150-00007

[B6] Shiffman RN Liaw Y Brandt CA Corb GJ Computer-based guideline implementation systems: a systematic review of functionality and effectiveness J Am Med Inform Assoc 1999 6 2 104 114 10094063 10.1136/jamia.1999.0060104 PMC61349

[B7] Hunt DL Haynes RB Hanna SE Smith K Effects of computer-based clinical decision support systems on physician performance and patient outcomes: a systematic review Jama 1998 280 15 1339 1346 10.1001/jama.280.15.1339 9794315

[B8] Shojania KG Jennings A Mayhew A Ramsay CR Eccles MP Grimshaw J The effects of on-screen, point of care computer reminders on processes and outcomes of care Cochrane Database Syst Rev 2009 3 CD001096 19588323 10.1002/14651858.CD001096.pub2 PMC4171964

[B9] Osheroff JA Pifer EA JM T Improving Outcomes with Clinical Decision Support 2005 Chicago, IL: HIMSS

[B10] Kilbridge PM Welebob EM Classen DC Development of the Leapfrog methodology for evaluating hospital implemented inpatient computerized physician order entry systems Qual Saf Health Care 2006 15 2 81 84 10.1136/qshc.2005.014969 16585104 PMC2464830

[B11] Neilson EG Johnson KB Rosenbloom ST Dupont WD Talbert D Giuse DA Kaiser A Miller RA The impact of peer management on test-ordering behavior Ann Intern Med 2004 141 3 196 204 15289216 10.7326/0003-4819-141-3-200408030-00008

[B12] Koppel R Metlay JP Cohen A Abaluck B Localio AR Kimmel SE Strom BL Role of computerized physician order entry systems in facilitating medication errors Jama 2005 293 10 1197 1203 10.1001/jama.293.10.1197 15755942

[B13] Bates DW Kuperman GJ Rittenberg E Teich JM Fiskio J Ma'luf N Onderdonk A Wybenga D Winkelman J Brennan TA Komaroff AL Tanasijevic M A randomized trial of a computer-based intervention to reduce utilization of redundant laboratory tests Am J Med 1999 106 2 144 150 10.1016/S0002-9343(98)00410-0 10230742

[B14] Thomas RE Croal BL Ramsay C Eccles M Grimshaw J Effect of enhanced feedback and brief educational reminder messages on laboratory test requesting in primary care: a cluster randomised trial Lancet 2006 367 9527 1990 1996 10.1016/S0140-6736(06)68888-0 16782489

[B15] Degoulet P Marin L Lavril M Le Bozec C Delbecke E Meaux JJ Rose L The HEGP component-based clinical information system Int J Med Inform 2003 69 2-3 115 126 10.1016/S1386-5056(02)00101-6 12810117

[B16] Nies J Colombet I Degoulet P Durieux P Determinants of success for computerized clinical decision support systems integrated in CPOE systems: a systematic review AMIA Annu Symp Proc 2006 594 598 17238410 PMC1839370

[B17] Gilmour S Degenhardt L Hall W Day C Using intervention time series analyses to assess the effects of imperfectly identifiable natural events: a general method and example BMC Med Res Methodol 2006 6 16 10.1186/1471-2288-6-16 16579864 PMC1481564

[B18] Wagner AK Soumerai SB Zhang F Ross-Degnan D Segmented regression analysis of interrupted time series studies in medication use research J Clin Pharm Ther 2002 27 4 299 309 10.1046/j.1365-2710.2002.00430.x 12174032

[B19] Cochrane Effective Practice and Organisation of Care Group http://www.epoc.cochrane.org last access on March 6, 2008

[B20] Eccles M Grimshaw J Campbell M Ramsay C Research designs for studies evaluating the effectiveness of change and improvement strategies Qual Saf Health Care 2003 12 1 47 52 10.1136/qhc.12.1.47 12571345 PMC1743658

[B21] Ansari F Gray K Nathwani D Phillips G Ogston S Ramsay C Davey P Outcomes of an intervention to improve hospital antibiotic prescribing: interrupted time series with segmented regression analysis J Antimicrob Chemother 2003 52 5 842 848 10.1093/jac/dkg459 14563900

[B22] Aarts J Doorewaard H Berg M Understanding implementation: the case of a computerized physician order entry system in a large Dutch university medical center J Am Med Inform Assoc 2004 11 3 207 216 10.1197/jamia.M1372 14764612 PMC400519

[B23] Solomon DH Hashimoto H Daltroy L Liang MH Techniques to improve physicians' use of diagnostic tests: a new conceptual framework Jama 1998 280 23 2020 2027 10.1001/jama.280.23.2020 9863854

